# Nicotinic Receptor Alpha7 Expression during Mouse Adrenal Gland Development

**DOI:** 10.1371/journal.pone.0103861

**Published:** 2014-08-05

**Authors:** Lorise C. Gahring, Elizabeth Myers, Sierra Palumbos, Scott W. Rogers

**Affiliations:** 1 Salt Lake City VA Geriatric Research, Education and Clinical Center, Salt Lake City, Utah, United States of America; 2 Department of Internal Medicine, Division of Geriatrics, University of Utah, Salt Lake City, Utah, United States of America; 3 Department of Neurobiology and Anatomy, University of Utah, Salt Lake City, Utah, United States of America; LSU Health Sciences Center, United States of America

## Abstract

The nicotinic acetylcholine receptor alpha 7 (α7) is a ligand-activated ion channel that contributes to a diversity of cellular processes involved in development, neurotransmission and inflammation. In this report the expression of α7 was examined in the mouse developing and adult adrenal gland that expresses a green fluorescent protein (GFP) reporter as a bi-cistronic extension of the endogenous α7 transcript (α7^G^). At embryonic day 12.5 (E12.5) α7^G^ expression was associated with the suprarenal ganglion and precursor cells of the adrenal gland. The α7^G^ cells are catecholaminergic chromaffin cells as reflected by their progressive increase in the co-expression of tyrosine hydroxylase (TH) and dopamine-beta-hydroxylase (DBH) that is complete by E18.5. In the adult, α7^G^ expression is limited to a subset of chromaffin cells in the adrenal medulla that cluster near the border with the adrenal cortex. These chromaffin cells co-express α7^G^, TH and DBH, but they lack phenylethanolamine N-methyltransferase (PNMT) consistent with only norepinephrine (NE) synthesis. These cell groups appear to be preferentially innervated by pre-ganglionic afferents identified by the neurotrophin receptor p75. No afferents identified by beta-III tubulin, neurofilament proteins or p75 co-expressed α7^G^. Occasional α7^G^ cells in the pre-E14.5 embryos express neuronal markers consistent with intrinsic ganglion cells and in the adult some α7^G^ cells co-express glutamic acid decarboxylase. The transient expression of α7 during adrenal gland development and its prominent co-expression by a subset of NE chromaffin cells in the adult suggests that the α7 receptor contributes to multiple aspects of adrenal gland development and function that persist into adulthood.

## Introduction

Nicotinic acetylcholine receptors (nAChR) are expressed and participate in the normal physiological functions of many neuronal and non-neuronal cell processes. One nAChR subtype, alpha7 (α7), is of particular interest because of its distribution in tissues throughout the body including both parasympathetic neurons and non-neuronal cells such as keratinocytes and those from the hematopoietic system (e.g., [1]–[4]). The α7 receptor is also distinguished from other nAChRs because it can function as a homomeric receptor composed of five-identical subunits, it has an exceptionally high permeability to calcium, and in addition to the endogenous ligand acetylcholine or the addictive substance in tobacco products nicotine, it is also fully activated by choline [3]. Together this diversity of ligand sensitivity and calcium signaling place the α7 receptor in a position to impact upon a spectrum of tissue and cell-specific responses that are in part shaped by the current physiological and environmental conditions.

An ongoing subject concerns the potential for α7 to directly or indirectly modulate sympathetic catecholaminergic systems (e.g., [5]–[8]). In the CNS nicotinic receptors including α7 modify catecholamine release to modulate neuronal responses, including adrenergic systems [9]–[11]. There are also reports placing nicotinic receptors (including α7) into sympathetic pathways important to control of catecholamine release (e.g., [8], [12]–[17]). For example, the elegant measurements of α7 modulation of the baroreflex hyper-responsiveness through altering NE release independently of parasympathetic control [12] attests to both the local modulatory contribution that is possible, but also the difficulty in making such measurements. The expression of α7 by peripheral catecholaminergic sympathetic systems has been suggested to include chromaffin cells of the adrenal gland [15], [17]–[19], and chromaffin cell-based expression libraries provided the starting material that contributed to the discovery of this and other clones encoding nicotinic acetylcholine receptor (nAChR) subunits (see [3]). Many of these receptor subunits are expressed in both developing and adult adrenal structures, but reports of the status of α7 expression in the adult has been less clear and often contradictory [20]–[22]. In part this likely reflects the relatively low level of α7 expression in the adult tissue and the difficulties inherent to measurement of this receptor's expression which has led to an emphasis on other nAChR subtypes [15]. Nevertheless, there is evidence that α7 contributes to normal adrenal gland function, especially during prenatal and early post-natal development [13], [17], [23]. This includes modulation of vesicular release from these adrenal cells [24]–[26], although the identity and distribution of α7 expression in this organ requires further clarification.

We examined α7 expression in the mouse adrenal gland during development and into the adult using recently developed α7 reporter mice where an IRES-tauGFP reporter is produced as a bi-cistronic extension of the endogenous gene transcript (α7^G^; [27]–[30]). The application of the precision of homologous recombination to introduce this reporter assured that minimal perturbation occurred to the normal copy number, genomic context and receptor protein structure. This improvement in detecting α7-expression also eliminates issues pertaining to the sensitivity of detection and other confounding problems such as the often low sensitivity or poor reliability of antibodies to α7-protein (e.g., [31]). The findings reveal extensive expression of α7 in the developing adrenal gland that is limited to chromaffin cells. As the adrenal chromaffin cells mature, α7^G^ expression is diminished dramatically until after birth where it becomes restricted to a small group of tyrosine hydroxylase+/DBH+/PNMT- cells in the adult. Our results support the conclusion that α7 participates in both adrenal gland development and later in life in NE regulation.

## Materials and Methods

### Animals

The animals were housed and used in accordance with protocols approved in advance by the Institutional Animal Care and Use Committee at the University of Utah (Protocol Number (09-07003)). In all cases animals were maintained according to the Guide for the Care and use of Laboratory Animals of the National Institutes of Health. The results reflect measurements of no less than 10 animals from independent litters for each evaluation. Adults were 2–5 months of age.

### Histology and Microscopy

The immunohistochemical methods are as described [27]–[30]. For embryos (E), developmental staging was based upon identification of coital plugs (equal to E0.5). Embryos were rinsed with phosphate-buffered saline and fixed in PBS containing 2% paraformaldehyde (PFA) and 5% sucrose for four hours. After fixation, the tissues were cryoprotected with sucrose in PBS to a final of 30% sucrose, embedded in gelatin and serial sections of 10–12 µm thickness were prepared using a Microm EM550 microtome. Sections were mounted on Fisherbrand Frozen Tissue microscope slides and air dried for at least 15 minutes before performing immune-staining overnight at 4°C. Adult adrenal glands were removed from mice at the post-natal or adult indicated and processed thereafter, similar to embryos. Direct visualization of GFP in fixed material prepared in this protocol was not possible. Because of this, all measurements of GFP expression were made using immune-labeling with anti-GFP primary antibody.

**Figure 1:**
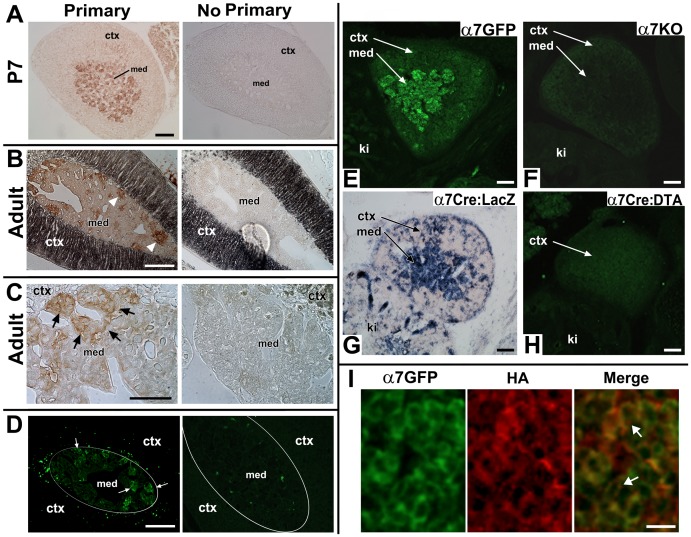
Specificity of α7^G^ detection in the adrenal gland. **A**. Transverse sections from a post-natal day 7 (P7) α7^G^ mouse adrenal gland. Anti-GFP (Primary) labeling using the immuno-peroxide method reveals cells localized throughout the adrenal medulla (med) that exhibit expression. Cells in the adrenal cortex (ctx) were not labeled. On the right is a similar section that was processed but the antibody to GFP was omitted (No Primary). **B**. Transverse sections from an adult α7^G^ adrenal gland processed for GFP immunoreactivity as in A. On the right an adjacent section was processed without addition of anti-GFP primary antibody. Arrow heads identify cells immunopositive for GFP signal which tend to be in clusters near the boundary of the medulla and cortex (darkly colored in this preparation). **C**. Increased magnification of the adult adrenal gland medullary region showing immune-reactivity towards GFP. The arrows point to cell clusters expressing GFP indicative of α7^G^ expression. The section to the right was again processed the same way but with omission of primary antibody. **D**. Transverse sections from an adult α7^G^ mouse adrenal gland were processed using immunohistochemistry to reveal anti-GFP immunofluorescence. On the right is a section processed without addition of anti-GFP primary antibody. Arrows identify α7^G^ cells immunopositive for GFP signal. The white oval outline approximates the boundary between the med and ctx. Note the similarity between the immunofluorescence pattern of GFP expression in cell clusters near the medulla-cortex boundary that closely resemble the pattern of immune-staining seen in B and C using the immuno-peroxide method. **E–H**. Additional tests confirm the specificity for measurement of the α7^G^ expression pattern in the adrenal gland. **E**. Sections were taken from E14.5 embryos of various genotypes related to α7 expression. First, immunofluorescence to α7^G^ (α7GFP) is revealed using and anti-GFP primary antibody. The anti-GFP signal is prominently expressed in chromaffin cells of the developing adrenal medulla (med), but it is essentially absent from the adrenal cortex (ctx). The kidney is identified (ki). **F**. A similar section was prepared from an α7 knock-out (α7KO) adult mouse and processed for anti-GFP immunofluorescence. No detectable GFP signal was observed. **G**. Another independent method to measure α7 expression was tested in an E14.5 embryo from the α7^Cre^ to a Rosa26-loxP(LacZ) reporter mouse cross (α7Cre:LacZ; for details see [28]). This embryo exhibits beta-galactosidase staining consistent with chromaffin cell expression. **H**. Sections prepared from E14.5 embryo of the α7Cre x Rosa26-LoxP(diphtheria toxin (DTA) cross to conditionally ablate (α7Cre:DTA; see [28]). Anti-GFP immunofluorescence detects no α7^G^ and there is no defined adrenal medulla (compare with E). **I**. In an E18.5 α7^G^ embryo the co-labeling for GFP (α7GFP; green) and the α7-associated hemagglutinin epitope tag (HA; see [28]) reveals GFP (green) that diffuses throughout the cytoplasm but does not enter the nucleus. The HA immunostaining (red) is located on the cell surface of the GFP expressing cells usually in a punctate pattern (identified by arrows). For A–H the Bar  = 100 µm and for I the Bar  = 30 µm.

All antibodies were commercially obtained. These were: dopamine-beta-hydroxylase (DBH; rabbit, 1∶1000, Abcam ab64953), glutamic acid decarboxylase 65/67(GAD65/67; rabbit, 1∶100, Sigma/Aldrich, G5163); neurofilament 68 (NF, mouse, 1∶1000, Sigma/Aldrich, G9670); neuronal nitric oxide synthase (nNOS; mouse, 1∶200, BD Transduction Laboratories, 610309), phenylethanolamine N-methyltransferase (PNMT; rabbit, 1∶150, Millipore/Chemicon, Ab110), tyrosine hydroxylase (TH; Rabbit, 1∶500, Millipore/Chemicon, Ab152 or mouse, 1∶1000, T2928); green fluorescent protein (GFP; chicken, 1∶800, Aves GFP-1020), beta-III tubulin (TUJ1; rabbit, 1∶3000; Covance, MMS-435P), hemagglutinin (HA; rabbit; 1∶200; HA.11 Covance, Princeton, New Jersey PRB-101P). Primary antibodies were incubated on sections overnight at 4°C. Sections were then washed and incubated for 1 hour at room temperature with appropriate secondary antibodies conjugated to peroxide or fluorescent markers ([28], Jackson ImmunoResearch). The sections were again washed, and either developed for peroxide staining (including pre-incubation with hydrogen peroxide to remove endogenous peroxide activity) or for fluorescence (immersed in anti-fade mounting medium before cover slipping and photography [28]). In all cases antibody specificity, either using peroxide visualization or immune-fluorescence, was confirmed on similarly prepared sections from mice either not modified by homologous recombination or where primary antibodies were not included during processing (see the Results and Figure 1 and [30]). Visualization of beta-galactosidase activity was as before [28]. In the adult adrenal gland non-specific autofluorescence in the green (FITC) channel proved limiting for visualizing GFP fluorochromes. To overcome this, it was necessary to detect GFP using other immune reporters (Jackson ImmunoResearch). However, for consistency with embryonic measurements, all images of GFP immunostaining from the adult were converted to green using Adobe Photoshop CS5 for use in the figures.

### Quantitative Analysis

Serial sections were prepared from adrenal glands of 3–6 embryos at each age. Alternating sections separated by 30–50 microns were stained for the identified markers. In each case 3–5 sections from the entire adrenal medulla were photographed at 20x magnification for both α7^G^ and the identified marker. These sections were then overlaid using Photoshop and the cells scored as α7^G^/marker negative or marker positive. In all cases plots and the mean and standard error of the mean were generated using either the Excel 2010 or GraphPad Prism 4 software.

## Results

The expression of α7^G^ in the adrenal chromaffin cell population was examined using both fluorescence and peroxidase-catalyzed diaminobenzidine deposition microscopy. The expression of α7^G^ in the adrenal gland, measured as immunoreactivity to GFP and detected by anti-GFP peroxidase coupled antibody, is shown in the post-natal (P) day7 (P7) mouse (Fig. 1A). Omission of primary antibody resulted in no signal (Fig. 1A). Similar results were obtained using immune-fluorescence microscopy (Fig. 1B) where again omission of primary antibody (FITC anti-GFP) resulted in no appreciable non-specific signal or immunostaining (Fig. 1B). The immune-staining pattern for α7^G^ becomes more restricted in the adult adrenal gland where anti-GFP primary antibody immune-staining signal is restricted to groups of cells that are largely localized to the perimeter of the adrenal medulla cortex and adjacent to the inner boundary of the adrenal cortex (Fig. 1C). Staining is absent in adjacent sections processed in parallel without primary antibody (Fig. 1C). At greater magnification (Fig. 1D), the expression pattern for α7^G^ is restricted to clusters of chromaffin cells localized near the adrenal medulla–cortex boundary. These are absent when primary antibody is omitted (Fig. 1D). Thus the detection of α7^G^ using these different methods produce the same result as expected from previously reported analyses of these mice [28]–[30].

Additional specificity of the α7^G^ immune-labeling was confirmed using genetically altered mouse strains (Fig. 1E). As shown for the E14.5 adrenal gland (returned to later) the expression of α7^G^ and α7^Cre^ [28] is restricted to immature chromaffin cell populations in the adrenal medulla. This staining is absent in the α7 knock-out mouse (α7^KO^, Fig. 1E) although the structure of the adrenal medulla and the presence of chromaffin cells appear normal in the α7^KO^ mouse. The expression of α7 during chromaffin cell development was also examined in the α7^Cre^ mouse in which the tau-GFP marker is replaced by a Cre recombinase [28]. The cell lineages identified by α7^Cre^ expression are measured in sections from embryos of crosses with the Rosa26-loxP(lacz) reporter mouse stained for beta-galactosidase activity [28]. The α7^Cre^xRosa26-loxP(lacz) cross embryos exhibit beta-galactosidase staining consistent with the α7^G^ expression (Fig. 1G). The α7^Cre^ mouse can be used to conditionally ablate α7-cell lineages in the embryos of crosses from mice harboring the Rosa26-loxP(diphtheria toxin (DTA)) mouse [28]–[30]. The results (Fig. 1H) show that in embryos of this cross there is near, if not a complete, loss of adrenal chromaffin cells with retention of the adrenal cortex. This also results in a coincident decrease in the adrenal gland size associated with loss of the α7^Cre^-ablated cells. Collectively, these results demonstrate by independent methods the specificity of the measurements used to measure α7^G^ expression.

To confirm that α7 expression coincides with the GFP reporter, we stained E18.5 chromaffin cells for both anti-GFP and the α7-associated C-terminal hemagglutinin epitope tag (HA; [27]–[30]). The results show (Fig. 1I) coincident immunostaining for GFP (which fills the cytoplasm but not the nucleus of the cell expressing α7^G^ and anti-HA. Because the HA tag directly measures α7 expression, it is located mostly in punctate clusters on the cell surface of anti-GFP reactive cells. There is excellent agreement between these markers of α7-expression. Immunostaining for GFP is preferred for measuring α7^G^ expression due to its ease of use and much greater signal/noise ratio.

### Expression of α7^G^ During Adrenal Gland Development

**Figure 2:**
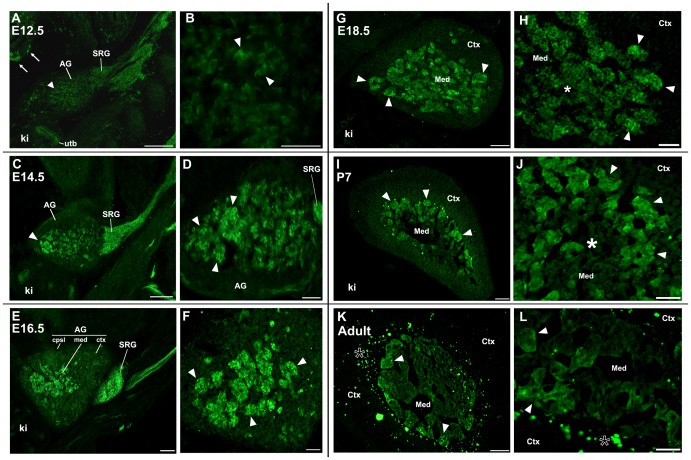
Expression of α7^G^ in the developing mouse adrenal gland. Sagittal sections of embryos at different stages of development were prepared and immunostained for α7^G^ (anti-GFP, Methods) to reveal current α7 expression. **A,B**. E12.5 embryos show the developing adrenal gland (AG) adjacent to the kidney (ki). Immunostaining for GFP is seen in the AG, present in the developing adrenal gland (AG, arrow heads), the suprarenal ganglion (SRG), enteric neurons (arrows) and the ureteric buds (utb) of the kidney. At increased magnification (B), the GFP signal in the AG and SRG reveals α7^G^ positive cells (arrow heads). **C,D**. The E14.5 AG begins to show consolidation of the cells expressing α7^G^ (arrow head) and ongoing expression in the SRG. At greater magnification (D), these cells tend to be in aggregates and often appear to be of increased density in the more ventral aspect of the AG (arrow heads). **E,F**. An E16.5 AG and associated SRG stained for GFP. At this stage α7^G^ expressing cells are in the medulla and few (if any) remain in the now recognizable capsule (cpsl) or ctx. Increased magnification (F) shows the majority of α7^G^ stained cells are in well-defined aggregates within the medulla (arrow heads). **G,H**. At E18.5 α7^G^ remains localized to well-defined cell groups in the med (arrow heads). The image on the right shows that at this stage there are also cells of reduced α7^G^ intensity (asterisk) relative to those identified by the arrow heads. **I,J**. The post-natal day 7 (P7) adrenal gland. Cells expressing α7^G^ tend to be aggregated into clusters that are localized towards the med-ctx border (arrow heads). This is particularly evident in (J) where the cell aggregates are visible while more centrally localized cells exhibit diminished or no α7^G^ expression (asterisk). **K,L**. The adult adrenal gland α7^G^ expression persisted in only a relatively few cells clustered into groups near the med-ctx boundary (arrow heads). The ‘spotted’ staining (asterisk) at this boundary interface and extending into the ctx is background fluorescence. Abbreviations: AG, adrenal gland; cpsl, adrenal capsule; ctx, adrenal cortex; ki, kidney; med, adrenal medulla; SRG, suprarenal ganglion; utb, ureteric bud. Scale bars  = 100 µm (A,B,C,E,G,I,K) or 50 µm (D,F,H,J,L).

The sympathoadrenal system arises from the ventral migration of the adrenal anlagen and includes mesoderm (fetal adrenal cortex) and chromaffin cells from the neural crest lineage [32]–[34]. We reported that the first detectable expression of α7^G^ in the embryo begins in rhombomeres 3 and 5 at embryonic day 9 (E9.0; [28]). Thereafter, α7^G^ expression increases throughout the embryo and exhibits considerable spatiotemporal patterning in a tissue-specific manner [27]–[30]. For example in the tooth organ, transient expression of α7^G^ occurs in some cells of the epithelium, mesoderm and again it returns to the enamel secreting ameloblasts of epithelium origin [32]. The first unambiguous expression of α7^G^ in the adrenal gland was at E12.5 (Fig. 2A). At earlier stages (E11.5), α7^G^ was inconsistently observed in anlagen-associated cells although strong labeling was seen in the suprarenal sympathetic ganglion (SRG) and sympathetic precursors (not shown). At the E12.5 stage α7^G^ is expressed by subsets of putative chromaffin cells that are dispersed throughout the developing structure (Fig. 2A,B). Also evident is the strong expression of α7^G^ in the SRG. Cells of the enteric nervous system and the ureteric buds of the kidney also express α7^G^ at this stage (Fig. 2B). The adrenal gland is encapsulated by E14.5 and the expression of α7^G^ is within cells contained within aggregates of cells that are centrally localized (Fig. 2C,D). The α7^G^ signal is not consistent across the structure as seen by the stronger expressing cells that are mostly towards the more distal aspect of the central aspect of the gland (Fig. 2D). The expression of α7^G^ cells are prominent in E16.5 adrenal glands where most are associated with centrally located aggregates that are consistent with the manifestation of boundaries that define the capsule, medulla and cortex (Fig. 2E,F). However, occasional α7^G^ cells are located outside of the central location of the forming medulla at this time (Fig. 2F). This is consistent with the retention of this portion of the adrenal gland subsequent to conditional ablation of the α7^Cre^ lineage that eliminated essentially all of the medullary compartment (see Fig. 1G). This pattern continues into E18.5 with further consolidation of the α7^G^ cells into aggregates that populate the central region of the adrenal gland consistent with the medulla (Fig. 2G,H). Overall there remains strong expression of GFP by many adrenal medullary cells although some appear to exhibit diminished α7^G^ signal (Fig. 2H). Only occasional α7^G^ cells (and often none) remain outside this central region at this stage (Fig. 2H). The expression of α7^G^ persists in the suprarenal sympathetic ganglion at this stage, but it is substantially reduced when compared to the earlier developmental stages (not shown). Signal in the kidney is absent (Fig. 2G) although expression by enteric neurons remains (not shown). After birth and by post-natal (P) day 7 (P7) the adrenal cortex and medulla are well defined (Fig. 2I,J). The expression of α7^G^ is restricted to chromaffin cells of the medulla and there is no immunostaining of cells in either the cortex or capsule (Fig. 2 I,J). Also at this stage the diminished expression of α7^G^ is clearly evident by the absence of signal in approximately half of the chromaffin cells which is most obvious in the more central aspect of the adrenal medulla. The diminishment of α7^G^ expression is particularly evident in the adult where signal becomes restricted to a smaller number of cells in aggregates near the border with the adrenal cortex (Fig. 2K,L). This pattern is also evident in the images from Figure 1C,D. The close association of α7^G^ chromaffin cells with each other and the tendency for these aggregates to be near the adrenal medulla periphery is apparent at greater magnification (Fig. 2L). This pattern persists into the adult.

### Developmental expression of the α7^G^ mouse by adrenal chromaffin cells and acquisition of catecholaminergic markers

**Figure 3:**
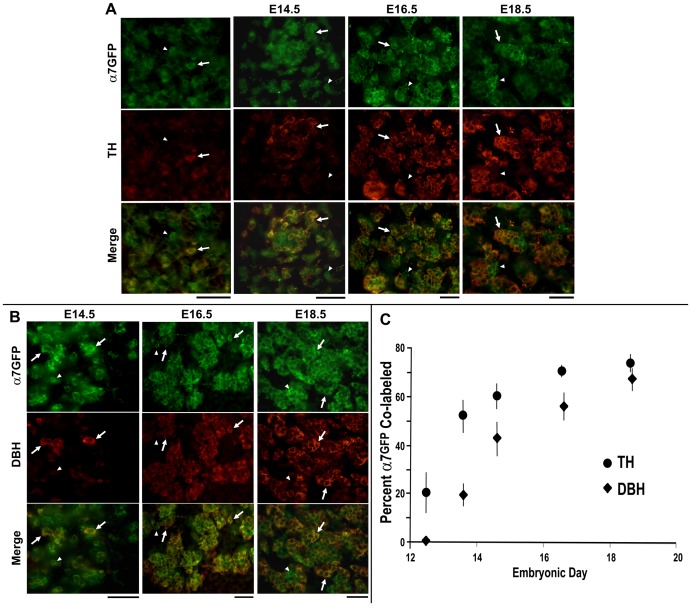
Coincident appearance of α7^G^ and catecholamine markers during chromaffin cell development. The adrenal glands from α7^G^ mice at the E12.5, E14.5, E16.5 and E18.5 developmental stages were co-stained for immunoreactivity to GFP (green) and in red either (A) tyrosine hydroxylase (TH) or (B) dopamine-beta-hydroxylase (DBH). **C**. Quantification of the α7^G^ cells co-labeled with either TH or DBH for the developmental stages. For each point at least 3–5 sections from the adrenal glands were collected from 3–6 different animals and the results from each individual animal were then summarized. The error bars reflect +/− standard error of the mean. Scale bar  = 25 µm (E12.5, E14.5) or 50 µm (E16.5, E18.5).

Adrenal function is divided into two distinct compartments [35]. The external cortex governs glucocorticoid production whereas the central region consisting of the medulla contains chromaffin cells that synthesize epinephrine and NE. The expression of α7^G^ by the two major types of chromaffin cells, adrenergic and noradrenergic, during development (E14.5 to E18.5) was examined. The distinction between adrenergic (epinephrine producing) and noradrenergic (norepinephrine (NE) producing) can be made using antibodies to detect the synthetic enzymes present that are required to generate these respective compounds. Three key activities detected by specific immune-reactivity are; tyrosine hydroxylase (TH; the rate limiting step for dopamine production), dopamine beta-hydroxylase (DBH; conversion of dopamine to NE) and phenylethanolamine N-methyltransferase (PNMT; the enzyme responsible for conversion of NE to epinephrine). The expression of α7^G^ coincident to either TH or DBH was measured and quantified in the adrenal gland of embryos from E12.5 thru E18.5 (Fig. 3).

During prenatal development the cellular expression of α7^G^ at E12.5 appears to precede most detectable TH expression (Fig. 3A). This is evident by the occurrence of a substantial number of α7^G^/TH-negative cells. Also at this stage are seen processes that express TH. This is consistent with early reports of the timing of sympathetic neuron appearance [36]. The discrepancy between α7^G^ and TH expressing cells persists into E14.5 embryos where essentially all TH cells co-express α7^G^ but only about half to the α7^G^ cells co-express TH (or are only weakly double stained; Fig. 3A). Again, this result suggests and is consistent with the onset of TH expression following α7^G^. Discrepancies between α7^G^ and co-labeling with TH persist at E14.5 although afterwards there is a progressive increase in α7^G^/TH co-labeled cells through E18.5 whereupon the maximum value of approximately 75% occurs. The expression of these α7^G^/TH-negative cells is returned to later. A similar result is seen for DBH although it trails TH presumably due to the delay in expression onset relative to TH (Fig. 3B,C). The percentage of α7^G^ co-labeled for DBH was consistent with an order of appearance being α7^G^, followed by acquisition of TH and then DBH.

### Adult adrenal chromaffin cells expressing α7^G^ co-express markers of norepinephrine synthesis

**Figure 4:**
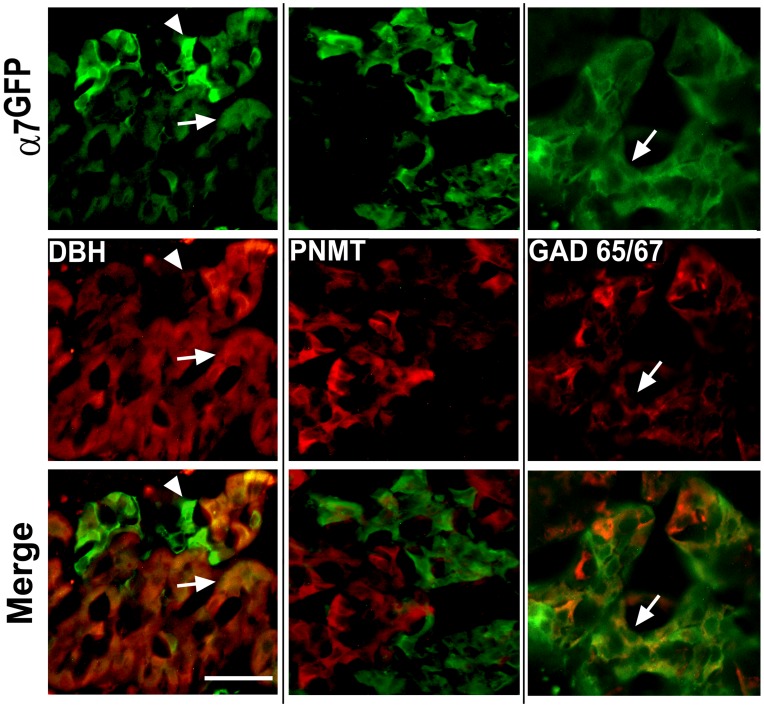
Co-expression of α7^G^, catecholamine markers and GAD65/67 in the adult adrenal medulla. Adult adrenal medulla immunostained for α7^G^ or DBH, PNMT or GAD 65/67 as identified. Cells expressing α7^G^ as measured by GFP immunofluorescence (green) are present and they tend to be clustered towards the adrenal medulla-cortex boundary (arrow). Some α7^G^ expression is localized to cells which are smaller and more solitary but exhibit a particularly strong GFP signal (arrow head). As shown most α7^G^ cells co-express DBH (arrow), but the more solitary cells do not (arrow head). In contrast co-expression of α7^G^ and PNMT has not been observed. The staining for GAD65/67 is largely discordant from that of α7^G^ although rare cells expressing both markers are identified (arrow). The extensive staining by GAD65/67 can also be found in some afferent fibers that are often associated with cells in clusters that express α7^G^ as is seen at greater magnification (cluster identified by the arrow). Scale bar  = 50 µm.

As was noted, α7^G^ expression in the adult becomes concentrated into cell groups that tend to be located near the adrenal-cortex boundary. These α7^G^ expressing cells also co-express catecholamine and additional markers (Fig. 4) that are distinct from those in the embryos. In the adult DBH is widely expressed by the cells of the adrenal medulla (Fig. 4A). This includes the majority, but not all, of the cells expressing α7^G^ (Fig. 4B). PNMT, while being absent in embryonic chromaffin cells (not shown), is also strongly expressed in the majority of chromaffin cells (Fig. 4B), but co-expression with α7^G^ is not detected. This is also consistent with the location of PNMT in more centrally located chromaffin cells and α7^G^ prevalence in the cell groups near the adrenal medulla outer perimeter. Collectively, these results provide evidence that adult α7^G^ chromaffin cells are of the phenotype; TH+/DBH+/PNMT-. This is also indicative that the expression of this nicotinic receptor is restricted to noradrenergic but not adrenergic catecholaminergic cell subtypes.

The identity of the remaining cells identified by strong α7^G^ expression is more complex. Some chromaffin cells synthesize and release GABA [37], [38] and these can be identified by the presence of glutamic acid decarboxylase (GAD). In the bovine adrenal gland approximately 30% of the chromaffin cells express GAD whereas the remainder expresses GABA receptors [39]. We examined the α7^G^ co-expression with GAD65/67 (Fig. 4C). The co-expression of these markers was seen in a subset of the total GAD65/67 staining cells (not shown) consistent with expectation that most GAD cells also synthesize epinephrine [39]. Thus, some overlap exists. The identity of additional cells that are often strongly labeled for α7^G^ (e.g., Fig. 4A) remains to be clarified.

### Innervation of the adrenal medulla

**Figure 5:**
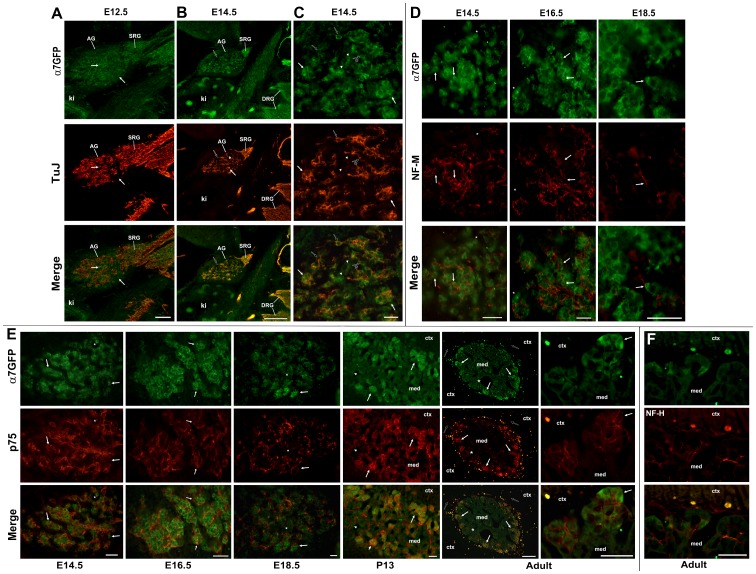
Adrenal gland innervation and expression of α7^G^. Adrenal glands at various times of development as indicated were labeled for co-expression of α7^G^ as measured by anti-GFP immunofluorescence (α7GFP, green) and various peripheral nerve markers as indicated (red). **A**) The same section from Figure 2a for the E12.5 adrenal gland is shown with co-labeling for immature neuronal marker, beta-III tubulin (TuJ). TuJ labeling reveals penetration of efferents into the developing adrenal gland (AG) and staining in the suprarenal sympathetic ganglion (SRG). With rare exceptions (arrows), there is almost no overlap in cells or processes expressing these respective markers. The kidney is identified (ki). **B**) The E14.5 AG exhibits consolidation of the TuJ staining into fibers that localize to the site of α7^G^ labeled cell accumulation. Rare cells are labeled by both α7^G^ and TuJ (black filled arrow). Note the coincident staining between these markers in the DRGs, but only very rare TuJ identified filaments in the kidney. **C**) Increased magnification of the E14.5 adrenal gland shown in B. In these images the cell exhibiting both α7^G^ and TuJ labeling is identified by the black arrow. Aggregated α7^G^ labeled cells with the presence of extensive TuJ-labeled afferents is identified for two of these clusters by arrows. Also present are α7^G^-labeled cells with no apparent association with TuJ labeled afferents (arrow heads). In some instances the TuJ identified afferents form fine fibers with varicosities that appear to wrap α7^G^ stained cells (black filled arrow with asterisk). **D**) Shown are images from the AG medulla at the developmental stage indicated double labeled for α7^G^ or neurofilament-M (NF-M). Arrows identify cells expressing α7^G^ that appear to also be in association with NF-M stained afferents. Note the consolidation and apparent trimming of NF-M afferents and their association with clusters of α7^G^ cells. **E**) Sections of the AG at the indicated developmental time are double labeled with α7^G^ and the neurotrophin receptor subunit p75. Although no co-labeling of cells or processes with α7^G^ and p75 was identified, there is extensive association between α7^G^ cells and p75 labeled afferents that persists into the post-natal period (P13 shown) and becomes refined to include almost preferentially cell clusters of the adrenal medulla identified by the α7^G^-labeled cell clusters in the adult (arrows). This is particularly evident in the adjacent panels showing these clusters at increased magnification. There are however, some cell clusters in the adult that exhibit weak or no α7^G^ immunostaining that are innervated by p75 stained fibers (asterisk). Occasionally p75-labeled processes also extend into the adrenal cortex (open arrow). These processes are not detected by anti-GFP staining. F) The adult immunostaining by the neurofilament protein-H (NF-H) exhibits less specificity for afferents interacting with α7^G^ stained cells when compared to those afferents identified by p75. Abbreviations: AG, adrenal gland; ctx, adrenal cortex; ki, kidney; med, adrenal medulla; SRG, suprarenal ganglion. Scale bar  = 100 µm (A,B, E adult) or 50 µm.

Adrenal control is regulated through complex signaling pathways that include hormonal modulation as part of its role in the hypothalamic-pituitary-adrenal axis, innervation by autonomic efferents and multiple elements that control local interactions [40]–[42]. The majority of innervation of the adrenal medulla and specifically that associated with chromaffin cells is from preganglionic cholinergic afferents as well as some innervation from a variety of other sources including somatosensory, possibly vagal influence via the splenic nerve and intrinsic neuronal processes [40]–[42]. The α7 nAChR is well-characterized to modulate multiple systems with responses as diverse as neurotransmitter release to pro-inflammatory immune responses [1], [3], [43]–[45]. Because of this important modulatory role and the possible expression of α7 by neurons, we examined the status of α7^G^ expression during innervation of the developing adrenal gland. To begin, early adrenal differentiation markers including both beta-III tubulin (TuJ) and neurofilament 68 (NF68) were visualized (Fig. 5). For the early neuronal marker TuJ (Fig. 5A), staining was already prominent at E12.5. Many of the adrenal associated TuJ-labeled processes associated with, and often appeared to wrap, the presumed chromaffin cells identified by α7^G^ (Fig. 5A; E12.5). In the E14.5 adrenal gland there is dramatic consolidation of the afferents identified by TuJ-labeling (Fig. 5B) including those processes projecting through the supra-adrenal ganglion and into the adrenal gland. The cell clusters identified by α7^G^ labeling are particularly seen to be associated with TuJ stained afferent fibers (Fig. 5B, C). This is very evident at increased magnification (Fig. 5C) where α7^G^ cells are in association with swellings on these TuJ-afferent terminals, which is characteristic of the varicosities, from which neurotransmitter is released. There are also solitary or small groupings of α7^G^ cells that appear to associate with only solitary fibers (Fig. 5C). In some more rare instances α7^G^ cells actually co-express TuJ (Fig. 5C). These could be intrinsic ganglionic cells of the adrenal medulla [46], but this remains to be confirmed. What is clear is that there is cellular heterogeneity identified by α7^G^ expression and the ability to assign a particular cell fate at this early stage of development is not guaranteed by the presence of this marker.

Innervation of the adrenal gland can also be distinguished by different neurofilament proteins (NF; [47]). We examined the pattern of staining for the three major NF forms (Fig. 5D) including NF-L (68 kD), NF-M (160 kD) and NF-H (200 kD). Similar to TuJ at E12.5, the expression of NF was dominated by NF-L which is more sparsely distributed and essentially absent from E14.5 embryos as reported (not shown; [47]). In the E14.5 gland, NF-M identified afferents that were largely in association with chromaffin cells expressing α7^G^ (Fig. 5D). Even at this early stage, the NF-M labeled fiber distribution suggests association with clusters of chromaffin cells expressing α7^G^. Also similar to TuJ staining many of these fibers appear to wrap α7^G^ cells (Fig. 5D). Nevertheless, α7^G^ identified as solitary or in small groups, are found in contact with NF-M labeled fibers. None of these fibers exhibit the GFP marker, which is prominent in axons of cells that express this marker due to the tau-partner on the GFP [28], [30]. By E16.5 NF-M labels afferents tracts that associate with ducts (not shown) but then become dispersed when in contact with α7^G^ chromaffin cell clusters (Fig. 5D). Another difference at this stage is the close association of these afferent fibers to the chromaffin cells seen at E14.5 is no longer present and terminal-like contacts are not common. In the E18.5 adrenal medulla the NF-M staining (Fig. 5D) is greatly diminished and restricted to only a few fibers that again associate with, an occasionally wrap, α7^G^ cells. Again, no α7^G^ and NF-M staining co-localized to the same process or cell.

Another strong marker of autonomic and somatosensory nerve fibers is the low-affinity neurotrophin receptor subunit p75 [48]. The expression of p75 receptor is present in the adrenal gland throughout development although it too did not co-localize with α7^G^ throughout the development of this gland (Fig. 5E). The expression of p75 was well defined in the developing adrenal gland at E14.5 where it could be observed in afferents intermixed throughout the α7^G^ labeled cells. As expected the pattern of expression closely resembled that seen for NF-M including the consolidation of fiber tracts in the E16.5 samples. By E18.5 p75-labeled fibers, unlike the NF-M, persist in afferent fibers associated with both cell clusters that express α7^G^ and those that do not. The persistence of p75 staining provides the ability to follow this innervation pattern into the postnatal mouse. During this time there is substantial consolidation of the fibers expressing p75 and the establishment of direct associations with chromaffin cells of the adrenal medulla that is clearly evident at P13.5 (Fig. 5G). The p75 afferents are not strictly associated with α7^G^ adrenal chromaffin cells, and by the adult (3 months) there are fiber terminations in association with both α7^G^-labeled and non-labeled cell clusters. None of these fibers co-express α7^G^ (Fig. 5E, adult). In the adult the p75 innervation pattern closely resembles that of afferent processes identified by NF-H immunostaining (Fig. 5F). In both cases α7^G^ cells exhibit contact with p75 and NF-H processes. Collectively this staining pattern suggest their identity to be pre-ganglionic sympathetic efferents [49] that preferentially innervate NE chromaffin cells.

## Discussion

Our study examines the temporal and spatial expression of nicotinic receptor α7 in the adrenal gland during embryogenesis, early post-natal development and in the adult. As in other organs, the expression of α7^G^ exhibits significant modifications throughout development [28]–[30]. The expression of α7 is present in cells that populate the adrenal gland at E12.5, very shortly after migration of the adrenal anlagen to its location adjacent to the kidney [36]. The α7G cells exhibit the progressive acquisition of catecholaminergic markers including TH and DBH suggesting that α7 expression begins in the cell precursor stage. There is a dramatic change in α7G expression during the post-natal period through weaning after which the adult expression pattern becomes restricted to adrenal chromaffin cells near the adrenal medulla margin that express TH and DBH, but not PNMT indicating these cells produce norepinephrine. Also observed is a smaller subset of these norepinephrine expressing chromaffin cells that co-express GAD65/67 [50].

The report of alpha-bungarotoxin binding by bovine adrenal medulla was an early indication of α7 expression by chromaffin cells [18]. The expression and specific role of α7 in chromaffin and adrenal gland function has since been examined in many systems although its specific contribution to the release of catecholamines and its expression by different cell types often varies between reports and possibly species. Our results suggest that the strongest α7 expression is by adrenal cells that are catecholaminergic and due to the absence of PMNT the catecholamine produced by these cells is norepinephrine. This result is in contrast to the earlier report of Criado et al. [20] who measured alpha-bungarotoxin in bovine chromaffin cells and found the majority of signal to correspond with cells also PMNT positive. However, similar to this earlier study we did find that chromaffin cells expressing α7 were located near the adrenal medulla-cortex boundary. Our results could differ for multiple reasons. First, this could simply reflect a difference in the species. For example, the relative chromaffin cell subtype ratio and possibly α7 expression itself varies substantially between reports in which different species are examined [51]–[54]. Second, α9/α10 receptors that can bind alpha-bungarotoxin are expressed in the bovine adrenal medulla and conditionally in the rat [55]. Thus, alpha-bungarotoxin binding does not necessarily offer exclusive specificity to α7 receptors. Third, the expression of α7 as measured by others [15], [20] is relatively low which is consistent with the co-expression and weak detection of the HA-epitope in this study. This could also complicate the detection of receptors using alpha-bungarotoxin. Finally, the expression of GFP from this α7 bi-cistronic genetic cassette might be sufficiently robust to obscure lower levels α7 expression in other cell types. Since mature α7 receptor numbers vary in terms of expression predicted by transcripts [3], this correspondence will require further investigation. Nevertheless, the strong expression of α7 but its limited distribution to only a relatively small population of NE chromaffin cells could be difficult to detect without the use of the more robust genetic-reporter methods used in this study.

Functionally the α7 receptor is particularly permeable to calcium [3] which is sufficient to contribute to the increase cytosolic calcium concentration in single chromaffin cells [60]. Under ideal conditions for receptor activation the calcium increase is sufficient to promote catecholamine release although it is not clear if this effect is through the direct calcium rise by α7 or possibly through a modulatory impact on voltage gated-calcium channels (e.g., [15], [17], [24], [52], [57]). Earlier studies [58], [59] showed that functional maturation of the neuronal-chromaffin synapse was associated with corresponding changes in post-natal α7 distribution and increased aggregation in the adult. From these results the investigators favored this effect to be related to an increase in the number of postsynaptic functional nAChRs in chromaffin cells [58], [59]. Our results are consistent with these studies since we also observe a significant redistribution of α7 expression in the early post-natal stages to the cells that compose the distinct peripheral chromaffin expression pattern observed in the adult. This includes aggregated expression of α7 on the cell surface of the NE-chromaffin cells examined. At the same time, rat chromaffin cells exhibit electrical coupling due to extensive connections by gap junctions that are in part under nicotinic cholinergic control including both α3β4 and α7. Hence, activation of α7 in one cell could have a more extended impact through impacting upon processes that are modulated through this coupling mechanism [52], [56], [58], [60] or the functional state of other associated cells could in turn modify the extent of α7 influence on any of these. Since the function of α7 is strongly modified by interactions with glucocorticoids [3], [61], the release of these agents from the adrenal cortex (particularly during times of stress) could participate in such an interaction as has been implicated by the of some sympathetic fibers and chromaffin cell functions to this hormone [62]–[64]. The well documented connections between nicotine and corticosteroids together with its role in neurotransmission [3], [5], [8], [61], [65], [66] collectively suggest the role of this receptor in modulating multiple physiological processes including the inflammatory response is likely to be widespread and be highly tissue specific.

Our findings regarding the post-natal afferent pattern is also in general agreement with previous studies examining the rapid change in expression and trimming of afferent cholinergic nerves in the post-natal adrenal gland [50] although we did not observe GAD staining of nerve fibers (not shown; [67]). The majority of innervation of the adrenal gland is by pre-ganglion sympathetic efferents that in early development can be identified by TuJ labeling or by NF-M. In the adrenal gland these processes do not co-label with α7^G^. In this regard, given the rich innervation of the adrenal gland from sympathetic pre-ganglion afferents, including those identified by p75 [49], and the favored termination by these afferents in α7^G^-NE chromaffin cell clusters; these afferents are strongly favored as the primary source of neuronal control of this interaction. In the p75-knock-out mouse noradrenergic function is specifically impaired despite otherwise normal cholinergic innervation and density [68]. Further, the baroreceptor response is regulated by adrenal norepinephrine release and regulation of different epinephrine and norepinephrine secretion [22], [69]. This is compatible with the participation by α7 in this basic sympathetic physiological function, as has been reported by others [8], [12]. Finally, in other tissues α7^G^ expression is detected in peripherin labeled afferents [29], [30], but this was not observed in the adrenal gland (not shown).

Others have reported that chromaffin precursor cell fate is sensitive to environmental conditions as demonstrated by studies showing significant impact on catecholamine production and secretion when embryonic chromaffin cells are exposed to nicotine or other conditions including hypoxia [70], [71]. In the case of nicotine exposure, repeated nicotine administration (1 mg/kg) leads to upregulation of TH and DBH, but not PNMT, in the adrenal gland [71]. As noted the function of the α7 receptor is remarkably sensitive to modulation by nicotine and dietary choline [3], [28]. Thus a consequence of developmental α7 pleiotropy is that maternal or early post-natal diet and exposure to agents delivering nicotine could ultimately impact on adult adrenal gland function in many different ways of varying severity and whose consequences that may not be realized until much later in life.
